# Supplementary material for: Mutations and Recombination at G4 DNA-Forming Sequences Exacerbated by CPT-Resistant Mutant Topoisomerase 1 Is Dependent on SUMOylation
Source: Int J Mol Sci. 2025 Sep 16;26(18):9017. doi: 10.3390/ijms26189017 (PMC12469889; doi:10.3390/ijms26189017)
Supplement: Supplementary file 1 [file ijms-26-09017-s001.zip › ijms-3856068-supplementary.pdf]

SUPPLEMENTAL MATERIALS

Table S1. Homologies Flanking Duplications in the DIPC2 mutation reporter.

# of homology (nt)	Sequence of homology	# Duplications in *top1*Δ	# Duplications in Top1Y740*
11	**GGGGAGCATGG**	10	3
7	**TGGGCGC**	0	4
8	**GGGGAGCA**	4	4
5	**TGGGG**	3	9
10	**GCGGGGAGCA**	24	19

The full sequences and details are in Figure 3A (*top1*Δ) and 3B (Top1Y740*).

Table S2. K65R, K91R, K92R mutation sequence.

WT	ACTAAGAAAATA**AAG**ACCGAACCAGTGCAATCGTCGTCATTACCATCGCCTCCA<br>GCAAAGAAAAGCGCGACATCAAAGCCTAAAAAAATC**AAGAAA**GAAGATGGTGA<br>TGTAAAGGTAAAA
RRR	ACTAAGAAAATA**AgG**ACCGAACCAGTGCAATCGTCGTCATTACCATCGCCTCCA<br>GCAAAGAAAAGCGCGACATCAAAGCCTAAAAAAATC**AgGAgA**GAAGATGGTGAT<br>GTAAAGGTAAAA

DNA sequences corresponding to AA61 to AA100 are shown. In WT, codons for K65, K91, and K92 are shown in Bold. In RRR mutant, the nucleotide mutations introduced are shown in lower case.

Figure S1

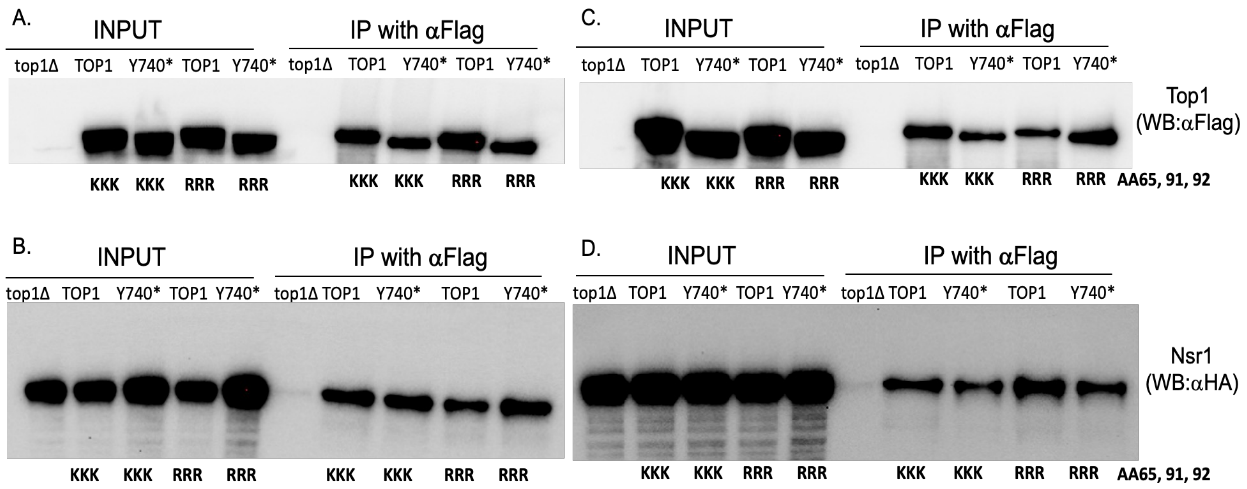

Figure S1. The effect of eliminating SUMOylation of Top1Y740* on Nsr1 interaction – additional experiments. Co-immunoprecipitation (co-IP) experiments conducted with whole cell extracts from *vtc4*Δ yeast strains expressing Top1-3XFLAG and Nsr1-6XHA. αFLAG antibody-coated agarose beads was used in pulldown. (A) and (B) are from one of three independent experiments were carried out and (C) and (D) are from another. See Figure 5 for more detail.
